# Waste Paper as a Valuable Resource: An Overview of Recent Trends in the Polymeric Composites Field

**DOI:** 10.3390/polym15020426

**Published:** 2023-01-13

**Authors:** Daniel Magalhães de Oliveira, Anne Shayene Campos de Bomfim, Kelly Cristina Coelho de Carvalho Benini, Maria Odila Hilário Cioffi, Herman Jacobus Cornelis Voorwald, Denis Rodrigue

**Affiliations:** 1 Fatigue and Aeronautical Materials Research Group, Department of Materials and Technology, School of Engineering and Sciences, São Paulo State University (UNESP), Guaratinguetá 12516-410, SP, Brazil; 2 Department of Chemical Engineering and CERMA, Université Laval, Quebec, QC G1V0A6, Canada

**Keywords:** waste paper, polymer, composite, recycling, mechanical properties

## Abstract

This review focuses on polymeric waste-paper composites, including state-of-the-art analysis with quantitative and qualitative discussions. Waste paper is a valuable cellulose-rich material, produced mainly from office paper, newspaper, and paper sludge, which can be recycled and returned to paper production or used in a new life cycle. A systematic literature review found 75 publications on this material over the last 27 years, with half of those published during the last five years. These data represent an increasing trend in the number of publications and citations that have shown an interest in this field. Most of them investigated the physicomechanical properties of composites using different contents of raw waste paper or the treated, modified, and cellulose-extracted types. The results show that polyethylene and polypropylene are the most used matrices, but polylactic acid, a biodegradable/sourced polymer, has the most citations. The scientific relevance of waste-paper composites as a subject includes the increasing trend of the number of publications and citations over the years, as well as the gaps identified by keyword mapping and the qualitative discussion of the papers. Therefore, biopolymers and biobased polymers could be investigated more, as well as novel applications. The environmental impact in terms of stability and degradation should also receive more attention regarding sustainability and life cycle analyses.

## 1. Introduction

Our world faces sustainable development challenges due to the scarcity of natural resources and the environmental impacts of all human activities. The 2030 Agenda for Sustainable Development consists of a plan guided by 17 sustainable development goals (SDG) to improve people’s lives now and in the future in a global partnership. SDG number 12 (responsible consumption and production) aims to achieve sustainable development through changes in consumption and production patterns through the efficient management of natural resources and changes in waste disposal through prevention, reduction, recycling, and reuse [1]. In 2020, 59.7% of the total amount of consumed paper was recycled in the world, mainly in Europe (73.3%) and North America (68%) [2]. Moreover, waste paper is considered the most recycled packaging in Europe (82%), followed by metals (77.4%), glass (75.4%), and plastics (40.6%).

Cellulose, a natural polymer extracted mainly from *Pinus* or *Eucalyptus*, is the raw material for paper production [3,4]. Paper is widely used in various products and purposes, such as printing, copying, packaging, and hygiene [5]. Once used, waste papers, mainly as office paper (printers, photocopiers, or drafts), old newspaper, and sludge paper (a byproduct of paper production), can become fuel sources destined for landfills or recycling. However, an enormous amount of waste paper (WP) is underused or inappropriately discarded. Approximately 40.3% of this paper is consumed, leading to global environmental problems [6]. Therefore, over the last 100 years, several studies have sought alternatives to increase the reusing or recycling of waste paper. Some examples are the production of valuable compounds, such as bioethanol [7,8] and activated carbon [9,10], as well as a filler in polymeric composites [11,12].

Natural fiber polymeric composites are increasingly used to manufacture more sustainable, resistant, and lighter materials with good specific properties and performance over a wide range of applications [13]. Because of their low cost, good performance, and ecofriendly attributes, waste paper from vegetal sources emerges as a potential substitute for (or combination with) synthetic fibers [14]. Several works reported that waste paper could be used as a filler for polymeric composites in the form of paper sheets for laminated composites [15], paper particles for injection molding [16], treated paper [17], modified with coupling agents [12], and cellulose extracted from the paper [18]. Their use improves the physicomechanical properties of the polymers and follows the environmental concepts of the 2030 Agenda (SDG).

This review aims to report and discuss what has been performed in terms of waste-paper valorization concerning polymeric composite applications. For this purpose, a systematic review was conducted using the two primary databases: Scopus and Web of Science. The analysis includes the number of publications, years, countries, keywords mappings, and the most cited documents. Furthermore, the leading composite polymers were identified, for which a bibliographic review was discussed under the document’s primary results, novelty, and findings. Finally, the openings for future works are given to guide the research and development of these composites and determine the gaps in the scientific literature about waste-paper polymeric composites.

## 2. Waste Paper: A Cellulose-Rich Material

**Figure 1:**
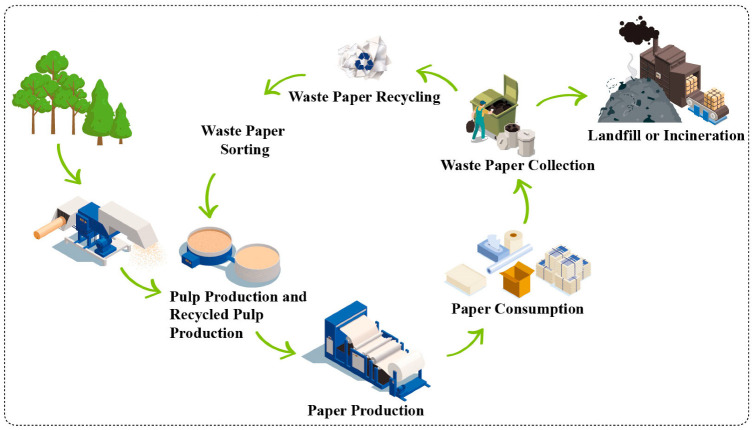
Paper production life cycle.

The paper production life cycle includes the extraction of the raw material from the natural resource (*pinus* and *eucalyptus* reforestation trees) or the raw material from the recycled waste paper: postconsumer-collected (Figure 1) [19]. The life cycle is closed and environmentally correct when paper recycling is included in the system. The raw material is transformed into a pulp that passes through a Kraft chemical process to remove lignin, followed by a bleaching process to remove other amorphous components, such as hemicellulose and the remaining lignin. Then, the paper production can be diverse, including office paper, newspaper, packaging, toilet paper, and cardboard. After the end-of-life of paper products, the waste is collected for landfill and incineration (initially) or recycling (more recently). Then, the recycled paper is sorted and returned into the “paper cycle” to produce a recycled pulp for paper production. Figure 1 shows the paper production cycle considering the paper recycling coming back for paper production, which should be achieved three-four times [20]. However, recycled paper can also be used in a new product life cycle, such as in composite materials or in producing valuable compounds (upcycling).

Table 1 presents the typical chemical composition of different types of waste paper, such as office paper, newspaper, and sludge paper. Among these, office paper is the most cellulose-rich waste-paper product due to its pulping (chemical processing), which removes most of the amorphous compounds. In contrast, newspaper generally goes through a different pulping process (mechanical or chemical/mechanical) in which a lower-strength paper is produced [19]. Paper sludge is a byproduct, including solid waste from pulp and paper production [21], generating waste paper with diverse chemical compositions.

Waste paper represents an old subject but still provides possible new developments. Recent articles reported waste paper uses for aerogels production finding different possibilities, such as spongy aerogels with oil absorption properties [27], superhydrophobic aerogel as a thermal insulating cooler for building fields [28], and an adsorbent aerogel to remove organic pollutants from wastewater (phenol and 2-chlorophenol) [29]. Additionally, the waste paper was converted to bioethanol [30], biodiesel [31], and biogas [32] through the action of micro-organisms. Other works synthesized the valuable compounds from waste paper, such as the biopolymer polyhydroxyalkanoate (PHA) by anaerobic digestion [33] and graphene from a carbonization process [34,35]. Furthermore, carboxymethyl cellulose (CMC) [36] and hydroxypropyl cellulose (HPC) [37] were synthesized via alkaline and ether reactions, while cyanoethyl cellulose (CEC) was synthesized via an alkaline reaction with acrylonitrile [38]. Packaging materials were also developed from waste paper alone with antimicrobial properties [39] or were mixed with other raw materials, such as silver nanoparticles [40] and sugar cane stalk + adhesives [41].

In addition to the applications mentioned above, the polymeric composites field has grown due to environmental issues and greater possibilities of using raw waste paper, treated waste paper, or cellulose-extracted waste paper, by varying the concentration and mixing methodology with different polymers (composites), as well as other fillers (hybrid systems). The following section will focus on polymer composites concerning the matrices used and the characterizations performed.

## 3. Waste Paper-Polymeric Composites

### 3.1. Systematic Review

Systematic research was performed using the Scopus and Web of Science databases, considering only documents (journal articles) in English. The repeated documents were excluded. First, the research included the words “*waste paper*”, showing a total of 4231 documents over the last 100 years (Table 2), discussing various applications, types of recycling, and the characterization of this residue. However, when the research was refined by combining the words “*waste paper*” and “*composite*”, the number decreased to 255 documents. The word “*polymer*” was not considered due to the different polymer names used in the abstract and title, such as polyethylene or polypropylene. Therefore, all the abstracts were reviewed, considering the ones related to the subject focusing on polymeric composites, leading to 75 documents.

**Figure 2:**
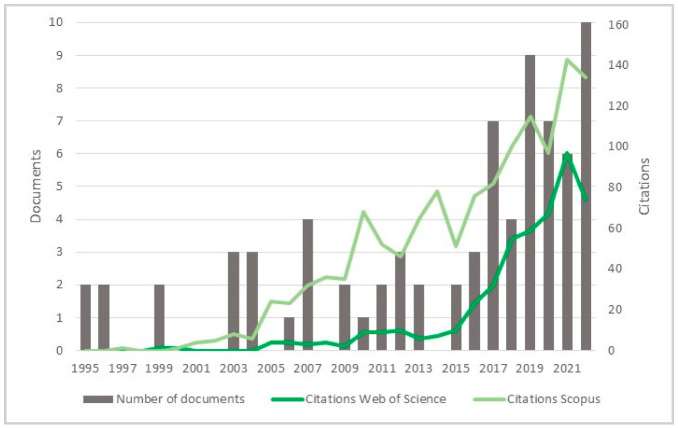
Number of publications and citations per year for the 75 documents, according to Scopus and Web of Science (1995–2022).

The 75 publications span the last 27 years (Figure 2). The data showed gaps along the timeline, but the subject has remained. Besides, an oscillation in publication numbers (increase and decrease) was observed over the years, but a general increasing trend was observed. The peak of publications was found in 2022 (incomplete year), followed by 2019, showing that this subject earned more attention in the recent scientific literature. Also, the number of publications was affected by the COVID-19 global pandemic in 2020 and 2021. Indeed, 50% of the volume of the documents was published over the last five years. Furthermore, the number of citations continuously increased over the years, becoming more relevant in 2006 as most were reported from Scopus, highlighting the scientific relevance of the subject.

**Figure 3:**
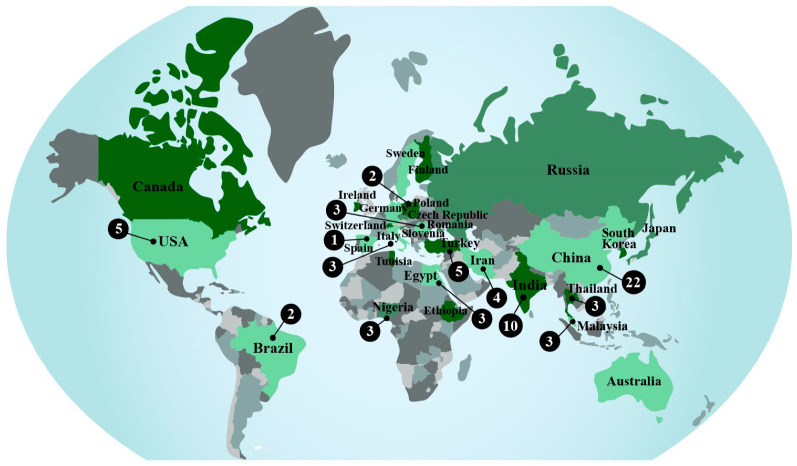
Number of publications per country in the 75 documents, according to Scopus and Web of Science (1995–2022).

Concerning geographic representation, 30 countries have published on the subject, with at least one from each of the five continents (Figure 3). The most represented country is China (22), followed by India (10), the USA (5), Turkey (5), and Iran (4), while the other countries have three or fewer documents. All these countries have less than 100 citations in their published documents, but the USA has the most cited paper in the scientific literature (Table 3). Also, collaborative research was identified among the countries sharing the same publication. For example, India with Ethiopia and Ireland [42], China with Switzerland [15], and Italy with Russia [43]. China has published on this subject over the last 20 years, while most countries have just started investigating the topic since half of the documents were published after 2017. Furthermore, over 50% of the world has not published on this subject yet since the publication list only includes 15% of the world’s countries. This indicates that the topic has started to be more widespread only recently.

**Figure 4:**
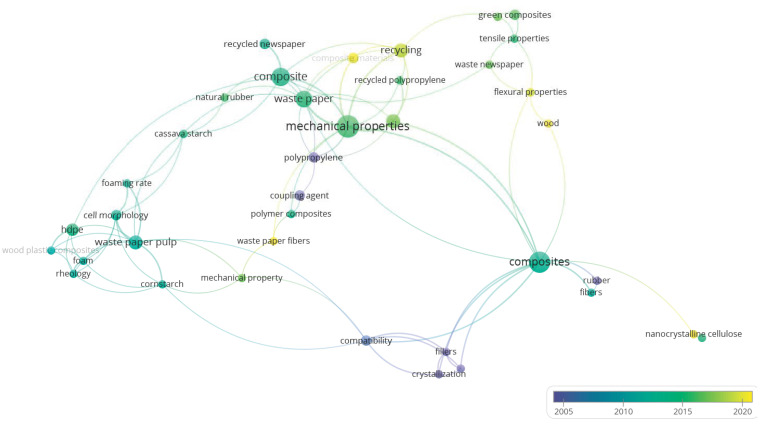
Keywords network in the overlay visualization mode, including frequency and chronological time from Scopus.

Keyword networking was performed using the software VOSviewer concerning the frequency and chronological time (Figure 4). The most incident cluster is *mechanical properties*, followed by *composite(s)*, *waste paper*, and *recycling*. Regarding chronological time, the yellow clusters are the most recent ones, including *recycling*, *wood*, *nanocrystalline cellulose*, and *flexural properties*. This result indicates the relevance of recycling and mechanical properties topics (including flexural properties). Other properties needed to be identified, suggesting a gap in the literature that could be explored more in further research.

The five most cited documents (Table 3) include articles published over ten years ago (1999, 2003, and 2006) from the USA, China, Iran, and India. The most important one (Huda et al. 2006) has two times more citations than the other four documents. Although it is not a recent article, it has been cited over the years until the present, confirming its influence and relevance in the literature (pioneering work).

Finally, it was possible to highlight the leading composites found in the 75 documents (Table 4). Conventional thermoplastics, thermosetting polymers, elastomers, and foams were identified. The highest number of documents was found for commodities polymers: polyethylene (PE) and polypropylene (PP) (also found in the keyword network). However, the biodegradable/sourced polylactic acid (PLA) provided a higher number of citations from all the published documents (438), including the most cited article from 2006 (Table 3) and more than six recent articles from the last five years (Table 4). Concerning the other biopolymers, one publication was found on PLA and PBAT (poly(butylene adipate-co-terephthalate)), but none were reported for other biopolymers. Therefore, these data support the scientific relevance of biopolymers and waste-paper-composite topics, indicating a scientific gap for new publications in the field of biopolymers as composite matrices.

Therefore, the subject has a low number of published documents (only 75) since it is a specific topic inside the outstanding waste-paper (WP) topic. So, there are apparent scientific gaps to be filled, highlighting several possibilities for new research on waste-paper-polymeric composites. The following section focuses on the different polymers used as matrices.

### 3.2. Main Polymers Used in WP Composites and Their Properties

Based on the systematic literature analysis presented above, it was possible to identify the main composite formulations discussed in the 75 documents. The following sections emphasize the main effects of adding WP to different polymers. A detailed discussion is included to support the systematic data found and better understand the gaps and prospects.

#### 3.2.1. Polyethylene (PE) Composites

The pioneering work reporting the influence of maleic anhydride coupling agent (5 wt.%) on the mechanical properties of low-density PE (LDPE) blended with high-impact polystyrene (HIPS) filled with WP (30 wt.%) presented a higher tensile (42%) and impact strength (38%), as well as a better dispersion of the composite’s filler with the coupling agent [48]. Other works also reported the mechanical behavior of PE/WP composites besides their thermal and morphological-structural properties. LDPE grafted with a maleic anhydride coupling agent filled with WP (10–50 parts per hundred—phr) has shown increased tensile strength (88%) and stiffness (409%) for a composite with 30 phr WP compared to a grafted polymer. This is supported by morphological analyses showing good interfacial adhesion between the polymer/filler, with fewer voids and pull-out [49]. Moreover, an interesting recent work used PE with WP (from beverage packaging) to produce a compressed laminated composite panel to replace typical wooden panels such as OSB (oriented strand board) [50]. The mechanical results presented an improvement in tensile strength (400%), tensile modulus (15%), three-point bending strength (54%), and three-point bending modulus (22%) for the composite with 50 wt.% PE compared to the OBS panel. The authors concluded that this material could replace wooden panels following environmental concerns.

In addition, PE was filled with extruded WP and tested for photochemical behavior after exposure to UV irradiation for 100 h [51]. The results showed a photo-oxidative degradation on the materials, justified by the formation of carbonyl groups (C=O), mainly for the composites with a higher WP concentration (20–30 wt.%) and, consequently, a reduction in crystallinity degree, compared to the composites with a lower WP concentration (5–15 wt.%). Additionally, all the composites presented lower thermal stability after UV exposure (around 20% for the 30 wt.% composites). Even with this result, the authors concluded that these composites could be applied to a wide range of applications where transparency is not required. Moreover, recycled LDPE from packaging films was mixed with WP in an extruder, compression molded and characterized via thermal and rheological analyses [52]. The addition of 40 wt.% WP slightly improved the thermal degradation temperature compared to the matrix (3 °C), while the other composites (50 and 60 wt.%) presented a decrease (4.5 °C) compared to the neat matrix. On the other hand, the complex viscosity increased as the amount of filler increased, presenting a more viscous material and, consequently, higher shear-thinning behavior.

Two kinds of WP (water-treated sludge and ink-eliminated sludge) at different concentrations (20–60 wt.%) were mixed with high-density PE (HDPE) with and without wood flour (WF) (20–60 wt.%) and were then extruded and injected [46]. In this hybrid system, both cellulose-based fillers act as reinforcements to improve the flexural properties of the composite. A Higher WF concentration increased the composite’s water absorption and thickness swelling. However, HDPE with 60 wt.% ink-eliminated sludge (WP) and a maleic anhydride coupling agent (3 wt.%) and without WF provided better physicomechanical properties compared to the other composites. In another work, HDPE, WP, WF, maleic anhydride coupling agent (MAPE), and starch-derived polymer using different compositions were mixed in a turbo mixer and compression molded [53]. The authors concluded that the composites with 10 wt.% WP, 30 wt.% WF and 3 wt.% coupling agents provided the best mechanical results regarding tensile strength and hardness.

WP was also mixed with pure cellulose and HDPE, followed by injection molding. However, the fillers were initially modified with fatty and amide acid esters (plasticizer), an aminosilane coupling agent, and a maleic anhydride coupling agent. The better interfacial adhesion between the components led to improved mechanical properties and moisture resistance, such as tensile strength for a composite modified with a maleic anhydride coupling agent (20%) [54]. Recycled HDPE was mixed and injected with WP deinking sludge. The results showed that adding the filler improved the crystallinity degree of the composites compared to the polymer since the filler acted as a nucleating agent [55]. In addition, a composite with 12.5 wt.% WP presented a higher tensile strength and stiffness than 5 and 20 wt.%, indicating that an optimum occurred, representing a balance between reinforcement and defects (dispersion, interface) with increasing filler content.

Novel research tested WP (5 wt.%) and chopped basalt (5 wt.%) in HDPE as a replacement for HDPE double-wall greenhouse glazing [43]. The injected composites showed a slight increase in melting temperature (4 °C) and tensile properties (8.2% and 11.4% in tensile strength and modulus, respectively) compared to the neat HDPE. They provided typical HDPE plastic behavior in the tensile test. This was considered an advantage since the material could be exposed to more extreme weather conditions, such as wind, snow, and rain, where plastic deformation, instead of fragile behavior, is required. Furthermore, a composite with HDPE, a 3 wt.% maleic anhydride coupling agent, and 40 wt.% WP (copy paper and poster paper) coated with CaCO_3_ was compressed and tested for water absorption with different CaCO_3_ concentrations (2.3, 4.6, 6.6, and 9.2%) [56]. After 14 days of water immersion, the composite with 9.2% CaCO_3_ provided the best behavior by decreasing its water absorption (from 5.3% to 2.5%) and thickness swelling (from 2.7% to 0.5%), indicating that calcium carbonate might be a good option (available and low cost) to improve the water-resistance of the HDPE/WP composites.

A recent study used HDPE and PP as matrices filled with WP from postage envelope waste (5–15 wt.%). The materials were extruded and injection molded into bars and discs. They found that the composites had increased dielectric losses: 40% for HDPE and 30% for the PP composites as the filler content increased since cellulosic fillers have polar OH groups compared to nonpolar polymers [57]. Another study used Xuan-WP from China as a filler for recycled PE/red mud composites. The composites were molded in a plate vulcanizing machine, and the results showed an improvement in the flexural strength (43%) but lower tensile strength (16%), especially with a higher (60 wt.%) WP content, while increasing the crystallinity degree of the composites [58]. This comes from the Xuan-WP containing minerals (27 wt.%) and organic particles (63 wt.%), mainly calcite, whewellite, and cellulose.

A novel piece of work developed a foam-compressed composite made of HDPE/WP using azodicarbonamide as a chemical blowing agent. The melt viscosity decreased, while the cell number increased (smaller cell sizes) as the blowing agent content (1–8 phr) increased. In contrast, a higher WP content led to higher melt viscosity, reducing the cell number and increasing the cell size [62]. Furthermore, HDPE/WP-injected composites were tested with different coupling agents: vinyltriethoxysilane (A151), vinyltrimethoxysilane (A171), and γ-methacryloxy propyl trimethoxy silane (KH570) [63]. A composite with 35 wt.% WP provided the best mechanical performance in terms of the improved tensile strength (19%), for which the modifier addition (3 wt.%), mainly A171 and KH570, generated a slight improvement not only in the mechanical properties but also in water absorption and thermal properties.

Finally, newspaper waste was tested as a WP filler (sheets) for HDPE (films)-laminated composites made from a hot-pressing process and characterized in a three-article series. The first one evaluated the relationship between flexural strength and composite density, as well as water absorption and porosity concerning WP content [15]. The flexural strength and density showed a linear correlation with maximum values of 99.4 MPa and 1.4 g/cm^3^, respectively, highlighting the influence of density on the mechanical properties of the composites. Water absorption was also correlated to porosity because the pores/voids in WP-filled HDPE are known to facilitate water penetration. The second work studied the influence of stair-like and vertical splicing on the properties of the HDPE/WP-laminated composites, in which the stair-like splicing provided the optimum physicomechanical behavior as the tensile strength increased (62% for 0° stacking direction to 11.1% for 90°) [59]. In contrast, the flexural properties decreased because of the poor interaction at the splicing position. The last paper focused on modifying the WP filler with stearic acid to improve the water resistance of the final composites [60]. The treatment was found to be effective in improving the composite’s water resistance, related to the esterification bonds between the WP hydroxyl groups and stearic acid carboxyl groups. All these interactions improved the tensile strength of the wet samples as the stearic acid concentration increased.

Table 5 presents an overview of the works published on WP-based PE composites for a quick reference and comparison of their properties.

#### 3.2.2. Polypropylene (PP) Composites

The effect of coupling agents on the properties of PP/WP was investigated in several works. Maleic anhydride grafted onto PP (MAPP) was compared with ethylene diamine dilaurate (EDD) as coupling agents in PP/WP composites that were extruded, and compression molded [12]. The results showed that EDD (2 wt.%) performed better by producing higher ductility (68%), flexural strength (19%), and impact strength (37%) but decreased the tensile modulus (10%) and water resistance (12%), with a similar tensile strength when compared to the same amount of MAPP. Another work investigated MAPP, stearic acid, and titanate as coupling agents in PP/WP composites that were extruded and injected [66]. It was found that MAPP generated the optimum interfacial interaction leading to a higher tensile strength (10%) and crystallization rate compared to the other compatibilizers. Moreover, MAPP (5 wt.%), as a coupling agent, was reported to increase the tensile strength (13%), flexural strength (70%), and impact strength (90%) compared to a composite without a coupling agent [45,68].

In addition, PP filled with WP, WF, and cellulose was hot-pressed and evaluated regarding water absorption. As expected, increasing the filler content (15–35 wt.%) increased the water absorption for all composites [44], but PP/WP presented the highest value, while PP/cellulose presented a higher water resistance. Furthermore, PP/WP (ink-eliminated sludge) was compared to PP filled with CaCO_3_, with both extruded and injection molded, showing that both fillers acted as nucleating agents in PP crystallization [67]. However, WP influenced the composite more since the melting and crystallization temperatures were higher for PP/WP than PP/CaCO_3_. The mechanical strength was better (about 20%) for PP with 30 wt.% WP compared to PP/CaCO_3_ with the same content.

A recycled PP mixed with WP as old newspaper (ON) and old magazines (OMs) was compression molded and showed the most significant properties and interfacial adhesion with 30 wt.% filler. However, WP-ON presented the highest mechanical properties for flexural strength (25%) [71]. Recycled PP filled with WP as newspaper (WP-N) was compared with better quality and more expensive filler: cellulose with a special bleaching pretreatment [69]. The results showed an improvement in tensile strength (26% and 56%) and stiffness (47% and 16%) for WP-N and cellulose, respectively, compared to recycled PP. At the same time, the thermal properties behaved similarly, with an increase in crystallinity degree for both the fillers. The authors concluded that cellulose performed better, but WP-N was more economically viable. In another work, recycled PP was mixed and hot-pressed with printed and unprinted WP to determine the effect of the ink on the material properties [70]. The presence of ink generated better interfacial interaction between the polymer and the filler due to its iron oxide content, thus improving the composite’s mechanical strength (tensile 37.9% and flexural 17.8%), tensile modulus (38.5%), and water resistance (25.5%).

An exciting work produced virgin and recycled PP filled with WP (from newspaper), nanoclay, and a coupling agent, for which the materials were put in a mixer rotating followed by injection molding [72]. The thermal and mechanical properties were slightly higher for the composites than the virgin PP composites. Nevertheless, the addition of nanoclay (2.5 wt.%) increased the thermal degradation temperature of the composites (32%), tensile strength (11%), and elastic modulus (23%) for PP/WP. The authors concluded that despite the slight decrease in properties for recycled PP, the values were sufficient for environmentally friendly applications, such as food utensils and automotive interior parts. In addition, PP was used with WP (newspaper) and glass fiber (GF) for outdoor applications in which the panels were hot-pressed. Water absorption and thickness swelling increased with filler content because the poor interaction between the materials generated a high number of voids/porosity facilitating water absorption [73]. However, higher water resistance with GF addition can open the door for wet locations, such as bathrooms and outside decks.

Another work evaluated the effect of four fillers (waste wood, kenaf core, waste jute, and WP newspaper) as reinforcement for injected PP composites to replace wood-plastic composites (WPC) [75]. The results indicated that any fillers could replace wood in the composites concerning mechanical properties. However, WP provided the best balance of properties. For example, WP improved flexural and tensile strength by over 25%. However, a more recent recycled PP and WP were produced to compare with cardboard or WF for 3D printing applications [64]. The composites were first extruded, and the specimens were 3D-printed to evaluate the physicomechanical properties. The recycled PP composites were compared to virgin PP composites, in which PP/WP (5–20 wt.%) did not improve the thermal and mechanical properties. But the addition of 10 wt.% WP in recycled PP generated a slight increase in the thermal stability (1.4 °C), glass transition temperature (36%), and tensile elastic modulus (25%), as well as a decrease in crystallinity degree (15%), and tensile strength (80%). Unfortunately, there was no conclusion regarding the feasibility of the material specified for 3D printing applications.

Conventional melt processing was replaced by solid-state shear pulverization (SSSP) via twin-screw extrusion to produce a PP/WP composite (15 wt.%) [65]. The composites were produced with different specific energy inputs (E_p_) to investigate their influence on filler size and dispersion. The results showed that medium to high E_p_ (14–35 kJ/g) provided a better filler dispersion, leading to an improved tensile elastic modulus (70 %), crystallization temperature (6%), and crystallinity degree (4%) at E_p_ = 14 kJ/g, compared to neat PP. Moreover, the cost of the composite was estimated to be lower than the neat PP, with both produced by SSSP. Another work investigated the thermomechanical performance of PP/WP composites made by injection, indicating that WP addition increased the stiffness and energy absorption capacity while decreasing the tensile strength and ductility [76]. However, 30 wt.% composites presented similar values to the neat PP, and 10 wt.% composites were suitable for nonstructural applications and for being more environmentally friendly by reducing the amount of synthetic material. Finally, a vintage article investigated the influence of WP particle size on the mechanical properties of PP composites with and without a MAPP wax coupling agent mixed by a K-mixer [77]. It was concluded that particle size did not influence mechanical strength, but MAPP provided an improvement of 26%.

Table 6 presents an overview of the works published on WP-based PP composites for a quick reference and comparison of their properties.

#### 3.2.3. Poly(Lactic acid) (PLA) Composites

PLA/WP composites (5–15 wt.%), with and without a silane coupling agent, were investigated as filaments for 3D printing [80]. The thermal results indicated a decrease in the thermal stability of the composites. However, the stability was still enough for 3D printing (190–210 °C), with a significant increase in crystallinity degree (134%). The tensile strength and ductility of the composites were improved with the addition of a silane coupling agent. Moreover, WP addition enhanced the melt flow properties of the composites, presenting higher shear-thinning behavior and fluidity than other well-known fillers, such as wood and cellulose nanocrystals.

Three sequential works from the same authors evaluated the influence of WP addition on PLA composites, as well as the influence of filler modifiers. The PLA/WP composites were compared with PLA/wheat straw and PLA/bamboo composites and showed optimum thermomechanical performance [11]. Furthermore, PLA with 20 wt.% WP and compatibilized with γ-(2,3-propylene oxide) propyltrimethylsilane (KH560) presented higher tensile strength (14%) and water resistance than the other composites. In subsequent work, nanocrystalline cellulose (NCC) was prepared from WP and mixed with PLA resulting in a composite with higher mechanical properties than neat PLA, such as tensile (8.2%), flexural (13.1%) and impact strength (35.9%) at 3 wt.% NCC [78]. Then, WP was mixed with a PLA/NCC composite and showed improved mechanical properties and water absorption compared to neat PLA, but this was still lower than PLA/NCC. According to the authors, the interfacial adhesion must be improved to produce higher mechanical properties in the composites, which were tested in more recent work. The influence of different coupling agents, such as γ-methacryloxypropyltrimethoxy silane (KH570), isopropyl tri(dioctylpyrophosphate) titanate, sodium hydroxide, poly-ethylene glycol 6000 (PEG6000), and a composite silane (KH570/PEG6000) was evaluated for PLA/NCC/WP composites [79]. From the microscopy images, it was possible to conclude that all the coupling agents provided interfacial interaction between the polymer and the fillers, enhancing the material’s properties by different degrees depending on the modifier. For example, the composites modified by KH570 presented higher thermal stability, while KH570/PEG6000 provided a higher tensile strength. Therefore, the best coupling agent depends on the properties required for the composite application.

The most cited article in the systematic analyses investigated PLA + WP (newspaper) and PLA + chopped glass fiber composites concerning their physicomechanical properties [16]. The main results showed an improvement in PLA/WP stiffness compared to neat PLA, identified by the increase in the tensile modulus, flexural modulus, and storage modulus. However, when considering PLA/glass fiber, the composites presented a slight reduction in almost all properties, except for glass transition temperature and crystallinity degree, which showed higher values in the PLA/WP composite. This little difference led the author to conclude that PLA/WP composites can replace PLA/GF composites in some applications, depending on the required properties.

A novel study evaluated the use of poly(butylene adipate-co-terephthalate) (PBAT) to improve the thermomechanical performance of PLA/WP composites [81]. PBAT addition (10–40 wt.%) promoted the impact strength (290%), thermal stability (4.1%), and crystallinity degree (10%) of the composites, compared to neat PLA. Furthermore, a coupling agent was added to improve the mechanical properties, leading to an optimum for PLA/WP with 20 wt.% PBAT and MAPLA/KH560 as compatibilizers. This result highlights the improvement of PLA performance by WP and PBAT for its use in nonconventional PLA applications that are different from packaging and 3D filaments. Another work evaluated the effect of treatment time (the filler went through a beating machine) and filler content on the properties of PLA/WP (corrugated paper) composites. As expected, WP addition (5–30 wt.%) increased the mechanical properties of the composite compared to neat PLA, such as their tensile (5–22%) and flexural (5–50%) strengths, while decreasing thermal stability (3–24%). The composite with 25 wt.% and 30 min of beating time provided the optimum mechanical performance, for which the authors concluded that this material could solve the environmental issues related to paper recycling [82].

Table 7 presents an overview of the works published on WP-based PLA composites for a quick reference and comparison of their properties.

#### 3.2.4. Rubber Composites

Rubber/WP (newspaper) composites were reported with enhanced mechanical properties compared to rubber/silica composites [17]. The composites were tested with natural rubber (NR) and butadiene acrylate copolymer rubber (NBR), with 20, 40, and 60% of the filler untreated and treated with sodium silicate and magnesium chloride. The results showed that rubber/WP composites with 40% WP provided the optimum mechanical properties, such as enhanced tensile strength (47.4%) and ductility (5.1%) compared to the silica composites. Moreover, the rubber/treated WP provided the best mechanical performance, especially for NR. Corn husk was used as a second filler for the rubber/WP composites to produce low-cost and low-weight composites with improved properties [84]. The results reported an improvement in composite wear resistance and water resistance as the corn husk content increased (2, 4, 6, 8, and 10 wt.%), which is associated with the hydrophobic nature of the corn husk. However, lower contents (4–8 wt.%) generated higher flexural properties. Rattan fibers (untreated and treated with KOH) were also investigated as a filler for NR/WP (pulp) composites and cassava starch/WP (pulp) composites. The results showed that cassava starch/WP/rattan composites provided higher flexural properties (~18%), while NR/WP/rattan presented a higher water resistance (~10%) [86]. The treatment improved the flexural properties while reducing the water resistance of all composites. Overall, the composites made with NR and WP provided the most outstanding water resistance without any treatments or other fillers (lower cost and easier to produce).

The fatigue life of two natural rubbers having 0 and 50 mole% epoxidations (SMR L and ENR 50) filled with WP (sludge) showed that increasing the WP content (10–40%) decreased the fatigue life of the composites. However, ENR 50 provided longer fatigue life [87]. Additionally, a maleated natural rubber coupling agent was used to increase the fatigue life of the composites by up to 14% at 30% WP and 22% at 10% WP.

Natural rubber was filled with WP (newspaper) that was modified with magnetite to generate radiation shielding, resulting in higher conductivity and mechanical properties than the neat matrix [88]. The NR/WP composites also included paraffin wax and boron carbide (B_4_C), which improved radiation shielding. The optimum was obtained at 42% modified WP, 18% paraffin wax, and 20% B_4_C. In recent work, NR + barium sulfate + WP composites were developed as a radiation attenuation material and were applied in X-ray shielding [85]. Prototypes with a 0.25 mm thickness were produced, showing good dispersion of the barium sulfate in the rubber matrix, and influencing the absorption of incident X-rays. Moreover, the maximum voltage of the X-ray beam for protection was 80 kV.

Table 8 presents an overview of the works published on WP-based rubber composites for a quick reference and comparison of their properties.

#### 3.2.5. Epoxy Composites

The first published work reported epoxy resin, phenolic resin, and WP (newspaper) composite laminates produced via prepreg, for which the mechanical performance was evaluated and compared to PP/WP [92]. The mechanical strength and modulus produced a decreasing trend as the filler content increased (0.3–0.7 wt.%), but the ductility was enhanced by 155% for the PP/WP 0.6 w.t% composite. The mechanical properties of the PP/WP composites were similar, such as tensile strength (56 MPa) for epoxy/phenolic/WP compared to 53 MPa for the PP/WP composites (for the same concentration). Another work produced laminate composites based on epoxy + WP (paper sheets) + woven jute, varying the stacking sequence and the layer content [91]. The WP composites provided the best mechanical properties compared to the jute composite and hybrid WP/jute composites, while the filler combinations (WP/jute) increased the stiffness of the composites. Electronic microscopy and acoustic emission analysis provided information on the failure mode of the composites during tensile and flexural tests.

Epoxy resin + WP composites were mixed with dammar natural resin (60–80%) and WP as paper sheets before and after shredding [90]. The results indicated that the tensile strength and modulus decreased with increasing dammar content. At the same time, the composites based on the WP sheets provided higher strength compared to the neat epoxy resin. The random distribution of the filler generated a lack of uniformity in the composite, where the tensile response was nonlinear for the shredded WP compared to linear for the WP sheets. Moreover, the damping properties of the material vibration were evaluated, showing an increase with enhanced dammar content, mainly for the composite with 80% dammar and shredded WP. The authors concluded that these hybrid composites based on shredded WP could be applied in the medical field as a material for fracture or sprain immobilization products. In contrast, hybrid composites based on WP sheets could be applied in the automotive industry for indoor products.

A recent work focusing on the environmental concerns caused by waste plastics developed a composite material from waste LDPE, WP, and epoxy resin [61]. Three formulations were evaluated, keeping the epoxy content (40%) but varying the WP (20–30%) and LDPE (30–40%). The results indicated that 20% WP and 40% LDPE improved tensile strength (17%) and water resistance (70%), while 25% WP and 35% LDPE improved flexural properties (15%) and stiffness (145%). Another work investigated the surface printability of the epoxy or polyester + WP (sludge) or cardboard waste composite plate [89]. The prints were made with water, solvent- and UV-based inks for a single color (cyan). The solvent-based and UV-based inks provided permanent surface adhesion, mostly observed for the WP composites, because of better ink adhesion than the cardboard composites. Epoxy/WP composites presented higher density than the polyester-based and cardboard-based composites, for which the density value was most significant for the solvent-based ink print.

Table 9 presents an overview of the works published on WP-based epoxy composites for a quick reference and comparison of their properties.

#### 3.2.6. Polyester Composites

It was reported that a polyester resin was mixed with WP as newspaper and kraft, with and without biaxially knitted glass fiber, in which the WP-kraft composite presented higher tensile strength (29%), ductility (109%) and stiffness (8.5%) but a similar impact strength to the WP-newspaper composite [95]. As expected, the knitted glass fiber composite significantly improved both composites’ mechanical properties, such as WP-kraft composite tensile strength (133%). The authors stated that the WP-kraft behavior was related to its hydrophilic nature, promoting better adhesion with the polyester resin. The polyester resin and WP-as-newspaper (25, 33, and 48%) laminate composites were also produced via hand lay-up as a low-cost raw reinforcement [94]. The mechanical results showed that the tensile strength increased as the amount of WP increased in the fiber’s direction (44.5 MPa for the WP 25% composite and 68.6 MPa for the WP 48% composite). In contrast, tensile strength decreased as the amount of WP increased in the cross direction (22.9 for the 25% WP composite and 19.1 for the 48% WP composite).

Water swelling properties were investigated in polyester/WP composites (25, 34, and 50 wt.%), indicating that water absorption and thickness swelling increased as the amount of WP increased [93]. Moreover, the mechanical performance of dry and wet composites was tested, showing that all the mechanical properties were lower for wet composites, being more significant for the 50 wt.% WP composites with a reduction of 40% in tensile strength and 50% in stiffness. Another work from the same authors evaluated the jute addition on polyester/WP laminate composites [47]. They reported improved mechanical properties regarding the polyester/WP composite, mainly for the composite with two layers of jute and one layer of WP in contrast to the two layers of WP and one layer of jute (~20% enhancement for tensile and flexural strength).

Table 10 presents an overview of the works published on WP-based polyester composites for a quick reference and comparison of their properties.

#### 3.2.7. Polyurethane (PU) Composites

PU/WP composites have only been reported recently. The first work reported PU and polyester mixed with WP and rice hulls, considering 80 *v*/*v*% of filler from which the composite plates were produced by compression molding [96]. The results showed that PU/WP presented the best water resistance performance. Moreover, PU/WP provides the highest mechanical properties compared to the other composites and neat PU. Compared to neat PU, PU/WP has a higher tensile strength (550%), stiffness (5000%), and hardness (12.5%), indicating that the flexible elastomeric PU became stiffer with the filler addition. The following article extracted cellulose nanocrystals from WP (WP-CNC) and filled waterborne PU composites to produce a transparent film via sonication after PU synthesis in-situ during PU synthesis [98]. The interaction among the hydrogen bonds between PU and WP-CNC was stronger for in-situ PU/WP-CNC than for sonicated PU/WP-CNC. Thermal stability was also improved for all the composites but sonicated PU/WP-CNC presented the most significant value. The authors concluded that both composites are applicable in medical and biological areas.

Another work studied waterborne PU mixed with cellulose nanocrystals (CNCs) from WP to produce membrane composites with two soaking times in water (20 s and 5 min) [97]. The CNCs were of the same quality as commercial cellulose but with higher thermal stability. Consequently, PU/WP-CNC composite membranes presented higher thermal stability than PU/commercial cellulose, for which the longer soaking time influenced better thermal properties. Additionally, a newer work compared WP-CNCs with microcrystalline cellulose and cellulose from waste cotton as reinforcement for PU composites [99]. The cellulose from WP provided a higher aspect ratio, crystallinity degree, and thermal stability than other cellulose sources. The significant interaction between PU and WP-CNC improved the composites’ thermal properties and stiffness.

Table 11 presents an overview of the works published on WP-based PU composites for a quick reference and comparison of their properties.

#### 3.2.8. Polyvinyl Alcohol (PVA) Composites

PVA was filled with WP, wood dust, and sisal fiber keeping WP (25 wt.%) and PVA (10 wt.%) concentrations constant but varying the wood dust (20–40 wt.%) and sisal (25–45 wt.%) contents [42]. The density decreased as the amount of sisal fiber increased. At the same time, the flexural strength was optimum at 30 wt.% wood and 35 wt.% sisal. The water absorption result indicated that a higher volume of sisal caused more significant water absorption. However, the most water-resistant composite was 30 wt.% wood and 35 wt.% sisal, as with the flexural results.

A second work investigated the effect of WP filler on epoxy resin, PP, and PVA composites regarding water absorption performance. In this case, injection molding and casting produced two types of PVA (GF 4–86 and 4–88) [74]. The results showed that epoxy/WP (3 wt.%), PP/WP/MAPP (30 wt.% and 4 wt.%), and casting/molded 4–88 PVA/WP (30 wt.%) provided lower moisture content, mainly for the PP and PVA composites. The authors concluded that molded 4–88 PVA/WP was the best composite with the optimum production method, good interfacial adhesion between the polymer/filler, and high crystallinity degree.

Table 11 presents an overview of the works published on WP-based PVA composites for a quick reference and comparison of their properties.

#### 3.2.9. Other Composites

Although conventional, well-known, and general polymeric composites have already been discussed, other synthetic and natural polymers were noticed at least once in the literature. Most of them were recently studied and published, being the reference for future research. One interesting recent work reviewed the novelty of cellulose-based materials, including WP as a rich-cellulose source, mostly applied to polymeric composites to improve their properties [100].

Some works reported some results on composites made from cellulose (WP-extracted). The starch-based composite was filled with nanocellulose from WP and commercial nanocellulose, from which the mechanical properties increased as the filler content increased (5–20 wt.%). However, the values were lower than for the starch-based commercial nanocellulose composites [18]. Nanocellulose from WP was also mixed with a guar gum film composite, indicating that the 4 wt.% nanocellulose composites provided the highest mechanical behavior with tensile strength (172%) and elongation (101%), compared to the neat guar gum film but stiffness (154%) was better for the 10 wt.% nanocellulose composite [22]. In another work, the cellulose from WP was mixed with chitosan and methyl red to obtain a composite film. The cellulose was successfully tested as a substrate for a colorimetric sensor. It indicates that these composite films could be used to detect food spoilage [101]. Furthermore, a novel work used cellulose from WP as a filler for a polypyrrole/graphene composite for supercapacitor electrodes. The cellulose addition promoted a higher specific capacitance (318%), power density, and energy density than neat polypyrrole, being suitable as an electrolyte tank [102].

Carboxymethyl cellulose and sodium alginate were mixed with WP to develop a biodegradable film to replace conventional packaging films with good mechanical properties and water vapor permeability [103]. Additionally, cassava starch was used with WP/bamboo and WP/rice husk. The composites with increased WP content presented a lower density and higher compression strength, suitable for construction blocks [104]. Composites based on maleated PVA, natural rubber graft cassava starch, modified cassava starch, and natural rubber/cassava starch filled with WP/sugar cane stalk were compared using different contents [41]. The impact strength and hardness increased as the polymers increased, while the swelling ratio decreased, concluding that mixed natural rubber/cassava starch composite provided the best physicomechanical performance.

Polyethylene terephthalate-1,4-cyclohexanedimethanol ester (PETG) was also filled with WP modified with an alkyl-ketene-dimer (AKD) and silane coupling agents (KH 550, 560, and 570). The optimum filler content was found to be 10 wt.%, and KH 550 was the most efficient for improving the composite’s performance by enhancing the mechanical properties and water resistance [105]. Moreover, a superabsorbent composite was studied using polyaspartic acid (PASP) with WP and 2-acrylamide-2-methyl-1-propanesulfonic acid graft with copolymerized acrylic acid. The results showed that the composite presented a high water absorbance, water retention, acid/base resistance, and salt resistance, leading to an adaptation in different environmental conditions [106].

To complete this review, Table 11 presents an overview of the works published on WP-based PU, PVA, and others composites for a quick reference and comparison of their properties.

**Table 11:** Main mechanical properties found in the PU, PVA, and other composites.

Matrix	Filler	Modifier	Processing	Main Mechanical Properties	Reference
PU	WP (20 *v*/*v*%)	N/A	Hot-pressing	Tensile strength: 7.8 MPa Tensile modulus: 741 Mpa	[96]
PVA	WP/wood/ sisal (25:30:35 wt.%)	N/A	Compression molded	Flexural strength: ~7.8 Mpa	[42]
Starch	WP/rice husk (70:10 wt.%)	N/A	Compression molded	Compression strength: 202 Mpa Impact strength: 130 J	[104]
Starch	WP-cellulose (20 wt.%)	N/A	Hot-pressing	Tensile strength: 29.8 Mpa Tensile modulus: 1396 Mpa	[18]
PETG	WP (10 wt.%)	KH550 ^1^ (1 wt.%)	High-speed mixer and hot-pressing	Tensile strength: ~55 Mpa Flexural strength: ~41 Mpa	[105]
Cassava starch/rubber	WP/sugar cane (20 *w*/*v*%: 25 g)	N/A	Compression molded	Impact strength: ~0.1 J/mm Hardness (Shore D): ~66	[41]
Guar gum	WP-nano cellulose (4 wt.%)	N/A	Film-forming solution	Tensile strength: 6.3 Mpa Tensile modulus: 19.1 Mpa	[22]

^1^ silane coupling agent.

## 4. Prospects

The studies included in this review used WP in different ways. Therefore, future works should still investigate new polymeric matrices different from conventional synthetic polymers, for which the composite materials will cause minimal or no environmental impact. Nowadays, several discussions on evaluating the environmental impacts, environmental footprint, and circular economy are included in any new development. Although a life cycle assessment (LCA) of the end-of-life of WP was published [107,108], none were found for WP composites. Some studies stated that WP composites are environmentally friendly materials but use many synthetic and/or virgin polymers. At the same time, no report was found on their impacts during and after their lifetime. Investigating composites’ end-of-life (via LCA), such as recycling and degradation, would be highly valuable. In addition, recycled and biobased polymers must be tested, especially to account for biodegradable biopolymers to resolve some of the environmental issues.

WP was reported to be a raw material for PHA synthesis. Future works could be carried out using PHA from WP in composites filled with WP, leading to an integrated biodegradable material with 100% materials from WP. Also, PHBV could be used in WP composites and PU foams. WP, as a cellulose-rich material, could positively affect PU foams’ stiffness and porous structure in terms of cell size and density. Biobased PU should be a priority and NR for more elastic applications.

Finally, new applications should be considered for WP composites to support material characterization. Three-dimensional printing is an outstanding and costly technology based on commercial filaments from PP, PLA, or acrylonitrile-butadiene-styrene (ABS). These filaments can be filled with natural fibers to obtain positive effects. Therefore, WP composites could be made based on PP or PLA filaments, influencing the material cost and performance.

## 5. Conclusions

The recent trends in polymeric composites were revised and critically discussed. A systematic review was performed using the two most important databases in the field. A total of 75 documents were found and discussed, highlighting their main conclusions and novelties concerning WP composite properties and application possibilities. These data provided valuable quantitative information about the number of documents, countries, keywords, and citations, indicating the scientific relevance of the subject and the gaps for future research.

The systematic bibliographic review showed that petro-based PP and PE had been vastly reported with WP filler with good physicomechanical properties depending on the filler content. In contrast, PLA is a biobased polymer but presented fewer publications with much higher citations, highlighting the scientific relevance of the PLA/WP composites. The keyword analysis showed that mechanical properties and recycling are the most frequent clusters, which were found in the discussion of the papers (Section 3.2).

Therefore, WP is a valuable cellulose-rich material that can be used for several applications, positively affecting the composite field. It is generally used as a reinforcement to improve mechanical properties or as a filler for enhancing water resistance, rheological properties, and thermal properties. Although several works have already been published, there are some new possibilities to fill the gaps observed in this review, and this should be a source of many works in the near future.

## Figures and Tables

**Table 1:** Chemical composition of different kinds of waste paper. ^1^

Waste Paper	Cellulose (%)	Hemicellulose (%)	Lignin (%)	Ashes (%)	Extractives (%)	References
Office Paper	61.8	12.6	9.2	11.3	5.7	[22]
	79.2	3.5	2.0	-	-	[23]
Newspaper	37.2	20.3	34.5	1.5	-	[24]
	55.2	15.3	29.5	-	-	[25]
Paper sludge	30.5	11.8	3.1	43.7	0	[21]
	44.6	6.7	22.6	30.2	2.9	[26]

^1^ Due to the inaccuracy of the chemical composition methods, the total of components for each sample does not always add up to 100%.

**Table 2:** Results of the systematic search in the Scopus and Web of Science databases.

Terms	Web of Science	Scopus	Total of Documents	Years of Publication
“*waste paper*”	640	3591	4231	1923–2022
“*waste paper*” and “*composite*”	7	68	75	1995–2022

**Table 3:** The five most cited documents in the 75 documents, according to Scopus (2012–2022).

Article Title	Reference	Country	Journal	Citations ^1^	Evolution of Citations ^1^
Chopped glass and recycled newspaper as reinforcement fibers in injection molded poly(lactic acid) (PLA) composites: A comparative study	[16]	USA	Comp. Sci. Tech.	397	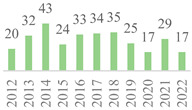
Water uptake and mechanical characteristics of natural filler-polypropylene composites	[44]	Iran	J. Appl. Pol. Sci.	108	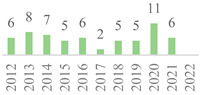
Maleated Polypropylene as a Coupling Agent for Polypropylene-Waste Newspaper Flour Composites	[45]	China	J. Appl. Pol. Sci.	61	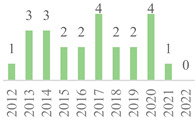
Effects of Waste Paper Sludge on the Physicomechanical Properties of High-Density Polyethylene/Wood Flour Composites	[46]	Iran	J. Polymer Envir.	59	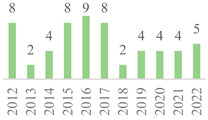
Mechanical properties of waste paper/jute fabric reinforced polyester resin matrix hybrid composites	[47]	India	Carbohydrate Polymer	53	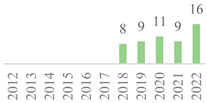

^1^ Until 30 September 2022.

**Table 4:** The main composites found in the 75 documents, according to Scopus and Web of Science.

Composites	Documents	Citations ^1^	References
Polyethylene + waste paper	19	148	[15,43,46,48,49,50,51,52,53,54,55,56,57,58,59,60,61,62,63]
Polypropylene + waste paper	17	351	[12,44,45,64,65,66,67,68,69,70,71,72,73,74,75,76,77]
Polylactic acid + waste paper	8	438	[11,16,78,79,80,81,82,83]
Rubber + waste paper	6	31	[17,84,85,86,87,88]
Epoxy + waste paper	6	55	[61,74,89,90,91,92]
Polyester + waste paper	6	98	[47,89,93,94,95,96]
Polyurethane + waste paper	4	44	[96,97,98,99]
Polyvinyl alcohol + waste paper	2	09	[42,74]

^1^ Until 30 September 2022.

**Table 5:** Main mechanical properties found in the PE composites.

Matrix	Filler	Modifier	Processing	Main Mechanical Properties	Reference
HDPE	WP (64.6 wt.%)	N/A	Hot-pressing (laminated)	Tensile strength: 101.5 MPa Flexural strength: 99.4 MPa	[15]
HDPE	WP (64 wt.%)	N/A	Hot-pressing (laminated-star-like splicing 0°)	Tensile strength: 109 MPa Tensile modulus: 9836 MPa Flexural strength: 119 MPa	[59]
HDPE	WP (78.5 wt.%)	Stearic acid (0.10 M)	Hot-pressing (laminated)	Tensile strength: ~65 MPa Tensile modulus: ~8500 MPa	[60]
HDPE	WP/chopped basalt (5:5 wt.%)	N/A	Extrusion and injection	Tensile strength: 17.1 MPa Tensile modulus: 641.8 MPa	[43]
HDPE	WP (60 wt.%)	MAPE ^1^ (3 wt.%)	Extrusion and injection	Flexural modulus: 3250 MPa	[46]
HDPE	WP/Wood (10:30 wt.%)	MAPE (3 wt.%)	Turbomixer and hot-pressing	Tensile strength: 27.7 MPa Tensile modulus: 1417 MPa Hardness Shore D (RT): ~70	[53]
HDPE	WP (60 wt.%)	MAPE + plasticizer (10:8 wt.%)	Extrusion and injection	Tensile strength: 42.1 MPa Tensile modulus: 2612 MPa	[54]
HDPE	WP/CaCO_3_ (40:9.2 wt.%)	MAPE (3 wt.%)	Extrusion and hot-pressing	Tensile strength: 18.3 MPa Flexural strength: 21.8 MPa	[56]
HDPE	WP (15 wt.%)	N/A	Extrusion and injection	Tensile strength: 4.6 MPa	[57]
HDPE	WP (35 wt.%)	KH570 ^2^ (3 wt.%)	Twin rotary mixer and injection	Tensile strength: 26.6 MPa Flexural strength: 37.8 MPa Flexural modulus: 2349 MPa	[63]
Recycled HDPE	WP (12.5 wt.%)	N/A	Extrusion and injection	Tensile strength: ~26 MPa Tensile modulus: ~1020 MPa Elongation at break: ~21%	[55]
Recycled HDPE	WP-Xuan/red mud (60:40 wt.%)	N/A	Plate vulcanizing machine	Tensile strength: 15.9 MPa Flexural strength: 71.8 MPa	[58]
PE	WP (50%)	N/A	Hot-pressing	Tensile strength: 12.9 MPa 3-point flexural strength: 23.8 MPa	[50]
LDPE + HIPS	WP (30 wt.%)	MAPE (5 wt.%)	Extrusion and injection	Tensile strength: ~17.5 MPa Tensile modulus: ~1.7 GPa Impact strength: 19.9 kJ/m^2^	[48]
LLDPE-g-MA	WP (30 phr)	N/A	Micro compounder and injection	Tensile strength: 7.9 MPa	[49]
Recycled LDPE + Epoxy (40:40%)	WP (20 wt.%)	N/A	Compression molded	Tensile strength: 10.1 MPa Tensile modulus: 422 MPa Flexural strength: 22.3 MPa	[61]

^1^ maleic anhydride graft polyethylene. ^2^ silane coupling agent.

**Table 6:** Main mechanical properties found in the PP composites.

Matrix	Filler	Modifier	Processing	Main Mechanical Properties	Reference
PP	WP (30 wt.%)	EDD ^1^ (2 wt.%)	Reomix polydrive and hot-pressing	Tensile strength: 29 MPa Flexural strength: 49 MPa Impact strength: 13.7 kJ/m^2^	[12]
PP	WP (15 wt.%)	N/A	Solid-state shear pulverization and hot-pressing	Tensile strength: ~32 MPa Tensile modulus: ~1700 MPa (Specific energy of 15 kJ/g)	[65]
PP	WP (30 wt.%)	MAPP ^2^ (5 wt.%)	Extrusion and injection	Tensile strength: 24 MPa Impact strength: 31.4 J/m Hardness: 66.8 N/mm^2^	[66]
PP	WP (20 wt.%)	N/A	Extrusion and injection	Tensile strength: 23.9 MPa Impact strength: 74.4 J/m Hardness: 62.4 N/mm^2^	[67]
PP	WP (30 wt.%)	MAPP (20 wt.%)	Extrusion and injection	Tensile strength: 29.9 MPa Impact strength: 51.6 J/m Hardness: 71.7 N/mm^2^	[68]
PP	WP (25 wt.%)	MAPP (2 wt.%)	Torque mixer and hot-pressing	Tensile strength: ~25 MPa Tensile modulus: ~2.1 GPa	[44]
PP	WP (50 wt.%)	MAPP (10 wt.%)	Two-roll mixer and hot-pressing	Flexural strength: 78.4 MPa Flexural modulus: 2916 MPa Impact strength: 17.1 kJ/m^2^	[45]
PP	WP (42 wt.%)	MAPP (5 wt.%)	Thermokinetic mixer and injection	Tensile strength: 48.9 MPa Impact strength: 27.2 J/m	[75]
PP	WP (30 wt.%)	N/A	Thermokinetic mixer and injection	Tensile strength: 24.5 MPa Tensile modulus: 1.1 GPa Elongation at break: 8.2 %	[76]
Recycled PP	WP (10 wt.%)	N/A	Extrusion and 3D printing	Tensile strength: ~19 MPa Tensile modulus: ~1450 MPa	[64]
Recycled PP	WP (40 wt.%)	MAPP + impact modifier (3:5 wt.%)	Extrusion and injection	Tensile strength: 29 MPa Tensile modulus: 564 MPa Elongation at break: 9.0%	[69]
Recycled PP	WP-ink office (50 wt.%)	N/A	Extrusion and hot-pressing	Tensile strength: 15.6 MPa Tensile modulus: 1.7 GPa Flexural strength: 26.9 MPa	[70]
Recycled PP	WP (30 wt.%)	MAPP (2 wt.%)	Double-roller mixer and hot-pressing	Tensile strength: ~32 MPa Elongation at break: ~11%	[71]
Recycled PP	WP/nano clay (30:2.5 wt.%)	MAPP (10 wt.%)	Mixer rotating and injection	Tensile strength: ~40.5 MPa Tensile modulus: ~1350 MPa	[72]

^1^ ethylene diamine dilaurate. ^2^ maleic anhydride graft polypropylenes.

**Table 7:** Main mechanical properties found in the PLA composites.

Matrix	Filler	Modifier	Processing	Main Mechanical Properties	Reference
PLA	WP-cellulose (30 wt.%)	N/A	Extrusion and injection	Flexural strength: 106.2 MPa Impact strength: 23.5 J/m Storage modulus: 10.1 GPa	[16]
PLA	WP (20 wt.%)	KH560 ^1^ (2 wt.%)	Rotary mixer and injection	Tensile strength: 58.9 MPa Flexural strength: 82.6 MPa	[11]
PLA	WP/nano cellulose (10:3 wt.%)	N/A	Extrusion and injection	Tensile strength: ~64 MPa Flexural strength: ~93.0 MPa Impact strength: ~10.5 kJ/m^2^	[78]
PLA	WP/nano cellulose (15:3 wt.%)	KH570 ^1^ + PEG6000 ^2^ (3:4 wt.%)	Extrusion and injection	Tensile strength: ~68 MPa Flexural strength: ~98 MPa Impact strength: ~11.5 kJ/m^2^	[79]
PLA	WP (10 wt.%)	KH570 (2 wt.%)	Extrusion and 3D printing	Tensile strength: ~50 MPa Elongation at break: ~6.2%	[80]
PLA	WP-corrugated (25 wt.%)	N/A	Reactor mixing and injection	Tensile strength: 29.5 MPa Tensile modulus: 907.3 MPa Flexural strength: 41.8 MPa	[82]
PLA + PBAT (68:20%)	WP (10 wt.%)	MAPLA + KH560 (2:2 wt.%)	Two-roll mixer and injection	Tensile strength: ~48 MPa Flexural strength: ~73.7 MPa Impact strength: ~15 kJ/m^2^	[81]

^1^ silane coupling agent. ^2^ poly-ethylene glycol 6000.

**Table 8:** Main mechanical properties found in the rubber composites.

Matrix	Filler	Modifier	Processing	Main Mechanical Properties	Reference
Rubber	WP (40 wt.%)	Na_2_SiO_3_ + MgCl_2_ (20 wt.%)	Two-roll mixer and hot-pressing	Tensile strength: 13.5 MPa Elongation at break: 850%	[17]
Rubber	WP/corn husk (70:8 wt.%)	N/A	Compacting machine	Flexural strength: ~0.9 MPa Flexural modulus: ~3.2 MPa	[84]
Rubber	WP-cellulose/ BaSO_4_ (60 wt.%)	N/A	N/A	Tensile strength: 11.2 MPa Elongation at break: 595%	[85]
Rubber	WP/rattan fiber (75:2)	KOH (1 M)	Compacting machine	Flexural strength: ~1.3 MPa Flexural modulus: ~38 MPa	[86]
Rubber (ENR 50)	WP (10 phr)	MNR ^1^	Hot-pressing	Fatigue life: 220 kc ^2^	[87]
Rubber	WP/paraffin/B_4_C (42:18:20 phr)	Magnetite (1:1)	Two-roll mixer and hot-pressing	Tensile strength: 7 MPa Tensile modulus: 1.9 MPa Elongation at break: 400%	[88]

^1^ maleic anhydride natural rubber. ^2^ kilocycles.

**Table 9:** Main mechanical properties found in epoxy composites.

Matrix	Filler	Modifier	Processing	Main Mechanical Properties	Reference
Epoxy	WP/dammar (60 wt.%)	N/A	Casting	Tensile strength 20.8 MPa Tensile modulus: 24.7 MPa	[90]
Epoxy	WP (37.5 wt.%)	N/A	Hand lay-up (laminated)	Tensile strength: ~92.5 MPa Flexural strength: ~121 MPa	[91]
Epoxy	WP (40 wt.%)	N/A	3-piece mold (laminated)	Tensile strength: ~56 MPa Tensile modulus: ~2.2 GPa	[92]

**Table 10:** Main mechanical properties found in the polyester composites.

Matrix	Filler	Modifier	Processing	Main Mechanical Properties	Reference
Polyester	WP (50 wt.%)	N/A	Hand lay-up (laminated)	Tensile strength: 70.2 MPa Interlaminar shear strength: ~7 MPa	[93]
Polyester	WP/Jute (42 wt.%)	N/A	Hand lay-up (laminated)	Tensile strength: 60 MPa Interlaminar shear strength: ~6 MPa	[47]
Polyester	WP (48 wt.%)	N/A	Hand lay-up (laminated)	Tensile strength: 68.6 MPa Tensile modulus: 5.9 GPa	[94]
Polyester	WP/biaxial glass fiber	N/A	Hand lay-up (laminated)	Tensile strength: 80.6 MPa Tensile modulus: 1424 MPa Impact strength: 130.1 kJ/m^2^	[95]

## References

[1] United Nations (2015). Transforming Our World: The 2030 Agenda for Sustainable Development.

[2] Cepi (2021). Monitoring Report 2021—European Declaration on Paper Recycling 2021–2030.

[3] Garrote G., Eugenio M.E., Díaz M.J., Ariza J., López F. (2003). Hydrothermal and pulp processing of Eucalyptus. Bioresour. Technol..

[4] Chinga-Carrasco G., Yu Y., Diserud O. (2011). Quantitative electron microscopy of cellulose nanofibril structures from eucalyptus and pinus radiata kraft pulp fibers. Microsc. Microanal..

[5] Campano C., Miranda R., Merayo N., Negro C., Blanco A. (2017). Direct production of cellulose nanocrystals from old newspapers and recycled newsprint. Carbohydr. Polym..

[6] Miranda R., Blanco A. (2010). Environmental awareness and paper recycling. Cellul. Chem. Technol..

[7] Dubey A.K., Gupta P.K., Garg N., Naithani S. (2012). Bioethanol production from waste paper acid pretreated hydrolyzate with xylose fermenting Pichia stipitis. Carbohydr. Polym..

[8] Annamalai N., Al Battashi H., Anu S.N., Al Azkawi A., Al Bahry S., Sivakumar N. (2020). Enhanced Bioethanol Production from Waste Paper Through Separate Hydrolysis and Fermentation. Waste Biomass Valorization.

[9] Sundriyal S., Shrivastav V., Kaur A., Dubey P., Mishra S., Deep A., Dhakate S. (2021). Waste Office Papers as a Cellulosic Material Reservoir to Derive Highly Porous Activated Carbon for Solid-State Electrochemical Capacitor. IEEE Trans. Nanotechnol..

[10] Shimada M., Hamabe H., Iida T., Kawarada K., Okayama T. (1999). The properties of activated carbon made from waste newsprint paper. J. Porous Mater..

[11] Zhang X., Li S., Xu C., Li J., Wang Z. (2020). Study on the mechanical and thermal properties of poly(lactic acid)/office waste paper fiber composites. J. Appl. Polym. Sci..

[12] Suryadiansyah, Ismail H., Azhari B. (2007). Waste paper filled polypropylene composites: The comparison effect of ethylene diamine dilaurate as a new compatibilizer with maleic anhydride polypropylene. J. Reinf. Plast. Compos..

[13] Hofstätter T., Pedersen D.B., Tosello G., Hansen H.N. (2017). State-of-the-art of fiber-reinforced polymers in additive manufacturing technologies. J. Reinf. Plast. Compos..

[14] Zhou Y., Fan M., Chen L. (2016). Interface and bonding mechanisms of plant fibre composites: An overview. Compos. Part B Eng..

[15] Zheng B., Hu C., Guan L., Gu J., Guo H., Zhang W. (2019). Structural characterization and analysis of high-strength laminated composites from recycled newspaper and HDPE. Polymers.

[16] Huda M.S., Drzal L.T., Mohanty A.K., Misra M. (2006). Chopped glass and recycled newspaper as reinforcement fibers in injection molded poly(lactic acid) (PLA) composites: A comparative study. Compos. Sci. Technol..

[17] Nashar D.E.E., Abd-El-Messieh S.L., Basta A.H. (2004). Newsprint paper waste as a fiber reinforcement in rubber composites. J. Appl. Polym. Sci..

[18] Takagi H., Nakagaito A.N., Bistamam M.S.A. (2013). Extraction of cellulose nanofiber from waste papers and application to reinforcement in biodegradable composites. J. Reinf. Plast. Compos..

[19] Rahman M.O., Hussain A., Basri H. (2014). A critical review on waste paper sorting techniques. Int. J. Environ. Sci. Technol..

[20] Kumar V., Pathak P., Bhardwaj N.K. (2020). Waste paper: An underutilized but promising source for nanocellulose mining. Waste Manag..

[21] Park H., Cruz D., Tiller P., Johnson D.K., Mittal A., Jameel H., Venditti R., Park S. (2022). Effect of ash in paper sludge on enzymatic hydrolysis. Biomass Bioenergy.

[22] Palanichamy P., Venkatachalam S., Gupta S. (2022). Improved recovery of cellulose nanoparticles from printed wastepaper and its reinforcement in guar gum films. Biomass Convers. Biorefinery.

[23] Lam D.N., Thien D.V.H., Nguyen C.N., Nguyen N.T.T., Van Viet N., Van-Pham D.T. (2022). Thermally stable cellulose nanospheres prepared from office waste paper by complete removal of hydrolyzed sulfate groups. Carbohydr. Polym..

[24] Woodward J., Stephan L.M., Koran L.J., Wong K.K.Y., Saddler J.N. (1994). Enzymatic Separation of High-Quality Uninked Pulp Fibers from Recycled Newspaper. Nat. Biotechnol..

[25] Mohamed M.A., Salleh W.N.W., Jaafar J., Asri S.E.A.M., Ismail A.F. (2015). Physicochemical properties of “green” nanocrystalline cellulose isolated from recycled newspaper. RSC Adv..

[26] Migneault S., Koubaa A., Riedl B., Nadji H., Deng J., Zhang S.Y. (2011). Binderless fiberboard made from primary and secondary pulp and paper sludge. Wood Fiber Sci..

[27] Pawar A.A., Kim H. (2022). Sustainable, hydrophobic, and reusable paper waste aerogel as an effective and versatile oil absorbent. J. Environ. Chem. Eng..

[28] Yue X., Wu H., Zhang T., Yang D., Qiu F. (2022). Superhydrophobic waste paper-based aerogel as a thermal insulating cooler for building. Energy.

[29] Pham T.H., Jung S.H., Kim Y.J., Kim T.Y. (2021). Adsorptive removal and recovery of organic pollutants from wastewater using waste paper-derived carbon-based aerogel. Chemosphere.

[30] Aghmashhadi O.Y., Asadpour G., Garmaroody E.R., Zabihzadeh M., Rocha-Meneses L., Kikas T. (2020). The effect of deinking process on bioethanol production from waste banknote paper. Processes.

[31] Nair A.S., Sivakumar N. (2022). Enhanced production of biodiesel by Rhodosporidium toruloides using waste office paper hydrolysate as feedstock: Optimization and characterization. Fuel.

[32] Rodriguez C., Alaswad A., El-Hassan Z., Olabi A.G. (2017). Mechanical pretreatment of waste paper for biogas production. Waste Manag..

[33] Al Battashi H., Al-Kindi S., Gupta V.K., Sivakumar N. (2021). Polyhydroxyalkanoate (PHA) Production Using Volatile Fatty Acids Derived from the Anaerobic Digestion of Waste Paper. J. Polym. Environ..

[34] Wang M., Liu C., Albolkany M.K., Zhao M., Zhu C., Liu B. (2021). Gram-Scale Synthesis of Porous Graphene via Printing Paper Pyrolysis as Supercapacitor Electrodes. Energy Technol..

[35] Singu D.C., Joseph B., Velmurugan V., Ravuri S., Grace A.N. (2018). Combustion Synthesis of Graphene from Waste Paper for High Performance Supercapacitor Electrodes. Int. J. Nanosci..

[36] He J., Wang D., Long L., Huang Y., Cui C., Yi J., Yang S., Wang Y. (2021). Preparation of Carboxymethylcellulose from Waste Paper. J. Wuhan Univ. Technol. Mater. Sci. Ed..

[37] Joshi G., Rana V., Naithani S., Varshney V.K., Sharma A., Rawat J.S. (2019). Chemical modification of waste paper: An optimization towards hydroxypropyl cellulose synthesis. Carbohydr. Polym..

[38] Joshi G., Naithani S., Varshney V.K., Bisht S.S., Rana V. (2017). Potential use of waste paper for the synthesis of cyanoethyl cellulose: A cleaner production approach towards sustainable environment management. J. Clean. Prod..

[39] Oliva C., Huang W., El Badri S., Lee M.A.L., Ronholm J., Chen L., Wang Y. (2020). Concentrated sulfuric acid aqueous solution enables rapid recycling of cellulose from waste paper into antimicrobial packaging. Carbohydr. Polym..

[40] Nwabor O.F., Singh S., Ontong J.C., Vongkamjan K., Voravuthikunchai S.P. (2021). Valorization of Wastepaper Through Antimicrobial Functionalization with Biogenic Silver Nanoparticles, a Sustainable Packaging Composite. Waste Biomass Valorization.

[41] Riyajan S.A. (2020). A packaging material from a waste paper/sugar cane stalk composite: Preparation and properties. Food Packag. Shelf Life.

[42] Gobikannan T., Berihun H., Aklilu E., Pawar S.J., Akele G., Agazie T., Bihonegn S. (2021). Development and Characterization of Sisal Fiber and Wood Dust Reinforced Polymeric Composites. J. Nat. Fibers.

[43] Sfarra S., Perilli S., Ambrosini D., Paoletti D., Nardi I., de Rubeis T., Santulli C. (2017). A proposal of a new material for greenhouses on the basis of numerical, optical, thermal and mechanical approaches. Constr. Build. Mater..

[44] Tajvidi M., Ebrahimi G. (2003). Water uptake and mechanical characteristics of natural filler-polypropylene composites. J. Appl. Polym. Sci..

[45] Yuan X., Zhang Y., Zhang X. (1999). Maleated Polypropylene as a Coupling Agent for Polypropylene-Waste Newspaper Flour Composites. J. Appl. Polym. Sci..

[46] Hamzeh Y., Ashori A., Mirzaei B. (2011). Effects of Waste Paper Sludge on the Physico-Mechanical Properties of High Density Polyethylene/Wood Flour Composites. J. Polym. Environ..

[47] Das S. (2017). Mechanical properties of waste paper/jute fabric reinforced polyester resin matrix hybrid composites. Carbohydr. Polym..

[48] Gatenholm P., Hedenberg P., Klason C. (1995). Recycling of Mixed Plastics Using Cellulosic Reinforcement. Plast. Rubber Pap. Recycl..

[49] Saini A., Yadav C., Bera M., Gupta P., Maji P.K. (2017). Maleic anhydride grafted linear low-density polyethylene/waste paper powder composites with superior mechanical behavior. J. Appl. Polym. Sci..

[50] Duşunceli N., Surme S. (2020). Mechanical Properties of Polymer-Matrix Cellulose-Based Composite Materials. Composite Materials: Applications in Engineering, Biomedicine and Food Science.

[51] Bajer K., Kaczmarek H., Dzwonkowski J., Stasiek A., Oldak D. (2006). Photochemical and Thermal Stability of Degradable PE/Paper Waste Composites Obtained by Extrusion. J. Appl. Polym. Sci..

[52] James A.R., Sbarski I., Masood S.H., Kosior E. (2007). Thermal and melt rheological behaviour of composites produced from waste paper and plastic. J. Polym. Eng..

[53] Valente M., Quitadamo A. (2017). Polymeric Matrix Composites at Reduced Environmental Impact. Polym. Eng. Sci..

[54] Merkel K., Rydarowski H. (2012). FTIR study and mechanical properties of cellulose fiber-reinforced thermoplastic composites. J. Biobased Mater. Bioenergy.

[55] Elloumi A., Makhlouf M., Elleuchi A., Bradai C. (2018). The potential of deinking paper sludge for recycled HDPE reinforcement. Polym. Compos..

[56] Peşman E., Tufan M. (2016). The effects of CaCo3 coated wood free paper usage as filler on water absorption, mechanical and thermal properties of cellulose-high density polyethylene composites. Medziagotyra.

[57] Ciobanu R.C., Batrinescu G., Ursan G.A., Caramitu A.R., Marinescu V., Bors A.M., Lingvay I. (2019). Mechanical and morphostructural characteristics of composite materials performed by recycling mixed waste of plastic and paper. Mater. Plast..

[58] Chen C., Yihe Z., Wanjia H., Cheng Q., Yongfan L., Na Z. (2020). Incorporation of Xuan-paper waste residue in red mud/waste polyethylene composites. J. Hazard. Mater..

[59] Zheng B., Guan L., Zhang W., Gu J., Tu D., Hu C. (2020). Production and characterization of large-scale recycled newspaper enhanced HDPE composite laminates. Polymers.

[60] Zheng B., Zhang W., Guan L., Gu J., Tu D., Hu C. (2021). Enhanced water resistance of recycled newspaper/high density polyethylene composite laminates via hydrophobic modification of newspaper laminas. Polymers.

[61] Shettigar Y.P., Kumar N., Aveen K.P., Hebbale A.M., Karinka S. (2022). Mechanical characterization of Waste Plastic and Waste Paper Composites (WPWPC). Mater. Today Proc..

[62] Zeng G.S., Xu C., Liu Y.J., Qu J.P. (2012). Rheological Behavior and Cell Morphology of Foamed Waste Paper Pulp/ High Density Polyethylene Composites. Appl. Mech. Mater..

[63] Zhang X., Di J., Xu L., Lv J., Duan J., Zhu X., Li X., Bo X. (2022). High-value utilization method of digital printing waste paper fibers-Co-blending filled HDPE composites and performance improvement. Polym. Test..

[64] Zander N.E., Park J.H., Boelter Z.R., Gillan M.A. (2019). Recycled Cellulose Polypropylene Composite Feedstocks for Material Extrusion Additive Manufacturing. ACS Omega.

[65] Iyer K.A., Lechanski J., Torkelson J.M. (2016). Green polypropylene/waste paper composites with superior modulus and crystallization behavior: Optimizing specific energy in solid-state shear pulverization for filler size reduction and dispersion. Compos. Part A Appl. Sci. Manuf..

[66] Qiao X., Zhang Y., Zhang Y., Zhu Y. (2003). Ink-eliminated waste paper sludge flour-filled polypropylene composites with different coupling agent treatments. J. Appl. Polym. Sci..

[67] Qiao X., Zhang Y., Zhang Y. (2003). Ink-eliminated paper sludge flour as filler for polypropylene. Polym. Polym. Compos..

[68] Qiao X., Zhang Y., Zhang Y. (2004). Maleic anhydride grafted polypropylene as a coupling agent for polypropylene composites filled with ink-eliminated waste paper sludge flour. J. Appl. Polym. Sci..

[69] Bogataj V., Fajs P., Peñalva C., Omahen M., Čop M., Henttonen A. (2019). Utilization of recycled polypropylene, cellulose and newsprint fibres for production of green composites. Detritus.

[70] Peşman E., Güleç T. (2019). The effects of ink presence on mechanical, physical, morphological and thermal properties of office and newspaper fiber-polypropylene composites. Medziagotyra.

[71] Xiaolin Z., Xiangfeng B., Rumin W. (2013). Study on mechanical properties and water absorption behaviour of wastepaper fibre/recycled polypropylene composites. Polym. Polym. Compos..

[72] Danesh M.A., ZiaeiTabari H., Hosseinpourpia R., Nazarnezhad N., Shams M. (2012). Investigation of the morphological and thermal properties of waste newsprint/recycled polypropylene/nanoclay composite. BioResources.

[73] Shakeri A., Ghasemian A. (2010). Water absorption and thickness swelling behavior of polypropylene reinforced with hybrid recycled newspaper and glass fiber. Appl. Compos. Mater..

[74] Vacková T., Kroisová D., Špatenka P. (2009). Water desorption kinetics of polymer composites with cellulose fibers as filler. J. Macromol. Sci. Part B Phys..

[75] Schneider J.P., Myers G.E., Clemons C.M., English B.W. (1995). Biofibers as reinforcing fillers in thermoplastic composites. J. Vinyl Addit. Technol..

[76] Cioffi M.G.A., de Oliveira D.M., de Bomfim A.S.C., de Carvalho Benini K.C.C., Cioffi M.O.H., Voorwald H.J.C. (2022). Thermo-Mechanical Properties of Polypropylene Composites Filled with Recycled Office Waste Paper. J. Nat. Fibers.

[77] Mölder T., Trass O. (1996). Grinding of waste paper and rice hulls with the Szego Mill for use as plastics fillers. Int. J. Miner. Process..

[78] Zhang X., Li S., Li J., Fu B., Di J., Xu L., Zhu X. (2021). Reinforcing effect of nanocrystalline cellulose and office waste paper fibers on mechanical and thermal properties of poly (lactic acid) composites. J. Appl. Polym. Sci..

[79] Zhang X., Di J., Li J., Li S., Duan J., Lv J., Zhu X., Xu L., Chang X. (2022). Effects of different interfacial modifiers on the properties of digital printing waste paper fiber/nanocrystalline cellulose/poly(lactic acid) composites. Polym. Eng. Sci..

[80] Tao Y., Liu M., Han W., Li P. (2021). Waste office paper filled polylactic acid composite filaments for 3D printing. Compos. Part B Eng..

[81] Xu C., Zhang X., Jin X., Nie S., Yang R. (2019). Study on Mechanical and Thermal Properties of Poly(Lactic acid)/Poly(Butylene adipate-co-terephthalate)/Office Wastepaper Fiber Biodegradable Composites. J. Polym. Environ..

[82] Su J., Jiang Z., Fang C., Zheng Y., Yang M., Pei L., Huang Z. (2022). The Reinforcing Effect of Waste Corrugated Paper Fiber on Polylactic Acid. Polymers.

[83] Levit M.R., Farrel R.E., Gross R.A., McCarthy S.P. (1996). Composites based on poly(lactic acid) and cellulosic fibrous materials: Mechanical properties and biodegradability. J. Eng. Appl. Sci..

[84] Oladele I.O., Abegunde O.O., Masud A.O. (2018). Flexural, water absorption and wear responses of green composites from bio-resources. Afr. J. Sci. Technol. Innov. Dev..

[85] Mungpayaban H., Rindhatayathon P., Ninlaphruk S., Rueanngoen A., Ekgasit S., Pengprecha S. (2022). X-ray protective materials from barium sulfate/amorphous cellulose/natural rubber composites. Radiat. Phys. Chem..

[86] Oluwole O.I., Avwerosuoghene O.M. (2015). Effects of cassava starch and natural rubber as binders on the flexural and water absorption properties of recycled paper pulp based composites. Int. J. Eng. Technol. Innov..

[87] Ismail H., Rusli A., Azura A.R. (2007). Study of fatigue life and filler interaction of paper sludge filled epoxidized natural rubber (ENR) and maleated natural rubber (MNR) composites. J. Polym. Environ..

[88] Madani M., Basta A.H., Abdo A.E.S., El-Saied H. (2004). Utilization of waste paper in the manufacture of natural rubber composite for radiation shielding. Prog. Rubber Plast. Recycl. Technol..

[89] Yüce H., Genç G., Sönmez S., Özden Ö., Akgül A., Çetiner B.N. (2022). Printability of bio-composite sheets made from paper mill and cardboard mill waste sludge. Pigment Resin Technol..

[90] Stănescu M.M., Bolcu D. (2020). A Study of Some Mechanical Properties of Composite Materials with a Dammar-Based Hybrid Matrix and Reinforced by Waste Paper. Polymers.

[91] De Rosa I.M., Santulli C., Sarasini F. (2011). Mechanical characterization of untreated waste office paper/woven jute fabric hybrid reinforced epoxy composites. J. Appl. Polym. Sci..

[92] Yadav P., Nema A., Varghese S., Nema S.K. (1999). Newspaper-reinforced plastic composite laminates: Mechanical and water uptake characteristics. Polym. Eng. Sci..

[93] Das S. (2017). Mechanical and water swelling properties of waste paper reinforced unsaturated polyester composites. Constr. Build. Mater..

[94] Das S., Basak S., Bhowmick M., Chattopadhyay S.K., Ambare M.G. (2016). Waste paper as a cheap source of natural fibre to reinforce polyester resin in production of bio-composites. J. Polym. Eng..

[95] Yenidoǧan S., Koçak D., Sesli Y., Sancak E. (2009). Investigation of mechanical, thermal and morphological properties of biaxial glass fiber/waste paper reinforced polyester composites. Sci. Eng. Compos. Mater..

[96] Calegari E.P., Porto J.S., Angrizani C.C., de Oliveira B.F., da Cunha Duarte L., Amico S.C. (2017). Reuse of waste paper and rice hulls as filler in polymeric matrix composites. Rev. Mater..

[97] Zhou X., Zhang X., Wang D., Fang C., Lei W., Huang Z., Song Y., He X., Huang Y. (2020). Preparation and characterization of waterborne polyurethane/cellulose nanocrystal composite membrane from recycling waste paper. J. Renew. Mater..

[98] Lei W., Zhou X., Fang C., Song Y., Li Y. (2019). Eco-friendly waterborne polyurethane reinforced with cellulose nanocrystal from office waste paper by two different methods. Carbohydr. Polym..

[99] Lei W., Pei H., Fang C., Zhou X., Zhang X., Pu M. (2022). Influence of nanocrystalline cellulose extracted from different precursors on properties of polyurethane elastomer composites. Compos. Sci. Technol..

[100] Motaung T.E. (2021). Recent applications and innovations of cellulose based materials: A critical review. Cellul. Chem. Technol..

[101] Phookum T., Siripongpreda T., Rodthongkum N., Ummartyotin S. (2021). Development of cellulose from recycled office waste paper-based composite as a platform for the colorimetric sensor in food spoilage indicator. J. Polym. Res..

[102] De Adhikari A., Oraon R., Tiwari S.K., Lee J.H., Nayak G.C. (2015). Effect of waste cellulose fibres on the charge storage capacity of polypyrrole and graphene/polypyrrole electrodes for supercapacitor application. RSC Adv..

[103] Kale R.D., Maurya Y., Potdar T. (2018). Paper-reinforced sodium alginate/carboxyl methyl cellulose-based bio-composite films. J. Plast. Film Sheeting.

[104] Yusuf T.A., Dan-Laaka L.E., Ogwuche P.O. (2017). Characterization of selected properties of composites of waste paper with untreated bamboo stem fibre and rice husk. Acta Polytech..

[105] Huang L., An S., Li C., Huang C., Wang S., Zhang X., Xu M., Chen J., Zhou L. (2018). Performance of waste-paper/PETG wood-plastic composites. AIP Adv..

[106] Sang W., Cui S., Wang X., Liu B., Li X., Sun K., Peng H., Ma G. (2022). Preparation and properties of multifunctional polyaspartic acid/waste paper fiber-based superabsorbent composites. J. Environ. Chem. Eng..

[107] Villanueva A., Wenzel H. (2007). Paper waste—Recycling, incineration or landfilling? A review of existing life cycle assessments. Waste Manag..

[108] Liu M., Tan S., Zhang M., He G., Chen Z., Fu Z., Luan C. (2020). Waste paper recycling decision system based on material flow analysis and life cycle assessment: A case study of waste paper recycling from China. J. Environ. Manag..

